# The pharmacokinetic interaction between nasally administered naloxone and the opioid remifentanil in human volunteers

**DOI:** 10.1007/s00228-021-03190-1

**Published:** 2021-07-29

**Authors:** Ida Tylleskar, Sissel Skarra, Arne Kristian Skulberg, Ola Dale

**Affiliations:** 1 Department of Circulation and Medical Imaging, NTNU – Norwegian University of Science and Technology, Trondheim, Norway; 2 Clinic of Emergency Medicine and Prehospital Care, St. Olav’s Hospital, Trondheim University Hospital, Trondheim, Norway; 3 Division of Prehospital Services, Oslo University Hospital, Oslo, Norway; 4 The Norwegian Air Ambulance Foundation, Oslo, Norway

**Keywords:** Naloxone, Naloxone-3-glucuronide, Intranasal administration, Opioid, Remifentanil, Drug interaction

## Abstract

**Purpose:**

Remifentanil has been shown to increase the bioavailability of nasally administered naloxone. The aim of this study was to explore the nature of this observation.

**Methods:**

We analysed samples from three pharmacokinetic studies to determine the serum concentrations of naloxone-3-glucuronide (N3G), the main metabolite of naloxone, with or without exposure to remifentanil. To enable direct comparison of the three studies, the data are presented as metabolic ratios (ratio of metabolite to mother substance, N3G/naloxone) and dose-corrected values of the area under the curve and maximum concentration (Cmax).

**Results:**

Under remifentanil exposure, the time to maximum concentration (Tmax) for N3G was significantly higher for intranasal administration of 71 min compared to intramuscular administration of 40 min. The dose-corrected Cmax of N3G after intranasal administration of naloxone under remifentanil exposure was significantly lower (4.5 ng/mL) than in subjects not exposed to remifentanil (7.8–8.4 ng/mL). The metabolic ratios after intranasal administration rose quickly after 30–90 min and were 2–3 times higher at 360 min compared to intravenous and intramuscular administration. Remifentanil exposure resulted in a much slower increase of the N3G/naloxone ratio after intranasal administration compared to intranasal administration with the absence of remifentanil. After remifentanil infusion was discontinued, this effect gradually diminished. From 240 min there was no significant difference between the ratios observed after intranasal naloxone administration.

**Conclusion:**

Remifentanil increases the bioavailability of naloxone after nasal administration by reducing the pre-systemic metabolism of the swallowed part of the nasal dose.

**Supplementary Information:**

The online version contains supplementary material available at 10.1007/s00228-021-03190-1.

## Introduction

The increasing number of deaths due to opioid overdose has been declared a public health emergency. This situation has led to an increased focus on opioid antagonism and the development of new antidote formulations, such as naloxone nasal sprays. Since 2015, four nasal naloxone products have been approved by medicinal regulatory authorities in Europe and the USA [1]. Approval was based only on pharmacokinetic studies in healthy volunteers.

Naloxone is a thebaine derivate with competitive opioid antagonistic properties. It has a terminal half-life of about 70—90 min. Its volume of distribution is about 200–300 L, and the clearance of naloxone is 3000—4000 mL/min [2, 3]. This value is considerably higher than the maximum liver clearance of approximately 1500 mL/min. This observation indicates that a considerable fraction of naloxone metabolism occurs in extrahepatic tissues. Moreover, the bioavailability of orally administered naloxone is only 2% [4, 5], indicating that naloxone is a high extraction drug. Naloxone is conjugated to its major metabolite naloxone-3-glucuronide (N3G), but n-dealkylated and reduced metabolites are also formed [4, 6, 7]. About 60% of the dose is excreted in the urine, the majority within 6 h [4].

Although naloxone has been used for decades, there is little knowledge on the pharmacokinetics of naloxone during exposure to opioid agonists, and only a few studies have evaluated opioid agonists and antagonists in combination [2, 8–11]. Skulberg et al. [2] used the bioequivalence criteria on data from two separate studies with the same nasal formulation, and observed that the area under the curve (AUC) of nasal naloxone was significantly higher in volunteers exposed to the opioid remifentanil [2] than in non-exposed subjects. In addition, the relative nasal naloxone bioavailability during remifentanil exposure was far higher than that described for other approved low-volume/high-concentration naloxone nasal sprays [12, 13]. Thus, a pharmacokinetic interaction between remifentanil and naloxone was hypothesised [2].

These observations prompted us to evaluate AUC values for naloxone (N-AUC) from our previous studies [2, 14–16]. We determined that the N-AUC_0–120_ increased by 13% for intravenous (IV) administration, 41% for intramuscular (IM) administration, and 65% for intranasal (IN) administration in remifentanil-exposed subjects compared to non-exposed subjects. The percentage increase in N-AUC_0_–_360_ was slightly lower compared to N-AUC after IN administration (Supplementary 1).

The nasal mucosa contains drug-metabolizing enzymes, not only phase 1 enzymes such as cytochrome P450 but also phase 2 enzymes such as glucuronosyltransferases (UGTs) [17]. We hypothesised that naloxone may be metabolised in the nose and that remifentanil exposure could inhibit the pre-systemic nasal metabolism of naloxone.

Interactions between naloxone and remifentanil and possibly other opioid agonists may have implications for future research and medicinal regulation as formulations of naloxone and other opioid antagonists as new nasal antagonist products are approved on basis of studies in healthy volunteers. We therefore decided to examine whether UGT-mediated formation of the main metabolite of naloxone, naloxone-3-glucuronide (N3G) [4], in our previous studies could support the hypothesis of pre-systemic nasal naloxone metabolism and whether remifentanil could act in this manner. To our knowledge this was the first study to examine the role of remifentanil on the metabolism of nasal naloxone.

## Material and methods

We analysed serum N3G in samples from healthy volunteers with or without exposure to remifentanil (remifentanil hydrochloride, C_20_H_28_N_2_O_5_) who were enrolled in three pharmacokinetic studies on naloxone (naloxone hydrochloride, C_19_H_22_ClNO_4_).

In study I, we investigated intranasal (0.8 mg) and intramuscular (0.8 mg) naloxone in healthy volunteers (n = 12) who were simultaneously exposed to the opioid remifentanil [2]. In study II, we investigated volunteers (n = 12) treated with 1.0 mg of intravenous naloxone while simultaneously receiving remifentanil infusion [15]. In studies I and II, remifentanil was administered as a target-controlled infusion 12 min before administration of naloxone and for another 90 min. In study III, we investigated intranasal naloxone (1.4 mg and 2 × 1.4 mg), intramuscular naloxone (0.8 mg), and intravenous naloxone (0.4 mg) in volunteers without co-administration of an opioid [16]. The third study included 22 participants; for the analysis of N3G, we randomly selected 12 participants due to resource constraints.

Nasal naloxone was manufactured by Department of Biopharmaceutical Production, Norwegian Institute of Public Health, Oslo, Norway for study I and by AS Den norske Eterfabrikk, Oslo, Norway for study III. The Aptar Unitdose device (Aptar Pharma, Louveciennes, France) was used. The formulation contained 8 mg/mL and 14 mg/mL naloxone hydrochloride in study I and III respectively, and the device delivered 0.1 mL per actuation. The nasal formulation have previously been published [14]. Naloxon B. Braun 0.4 mg/ml (Melsungen, Germany) was used for intravenous and intramuscular administration of naloxone. Remifentanil Ultiva 2 mg (GlaxoSmithKline, Brentford, UK) was used for the opioid infusion.

All studies were conducted in accordance with the Declaration of Helsinki and Good Clinical Practice (GCP). All protocols were approved by the Regional Committee for Medical and Health Research Ethics and the Norwegian Medicinal Authority. The studies were registered with the European Union Drug Regulating Authorities Clinical Trial database and ClinicalTrials.gov. The participants were insured by the Drug Liability Association, Norway. The design of each study is presented in Supplementary 1.

Samples for analysis of naloxone and N3G were collected before naloxone administration and at 2, 5, 10, 15, 20, 25, 30, 35, 45, 60, 90, and 120 min in all studies. In studies I and III, additional samples were collected at 240 and 360 min. Naloxone and N3G were analysed using a validated high-performance liquid chromatography tandem mass spectrometry method at the Proteomics and Modomics Experimental Core Facility (PROMEC), Norwegian University of Science and Technology, Norway [18, 19]. The analytical methods are described in detail in Supplementary Table 1.

The serum concentration data were analysed using non-compartmental techniques and Win-Nonlin version 8.0 (Pharsight Corporation, NJ, USA). The AUC was calculated from the first 20 min, for the first 120 min, and up to 360 min. The maximum concentration (Cmax) and time to maximum concentration (Tmax) were estimated using the same program. The metabolic ratio, the ratio of the AUC of the metabolite (N3G-AUC) to the AUC of the mother substance naloxone (N-AUC), was used to compare different dosing and administration regimes. The Cmax and AUC values were dose corrected to allow for direct comparisons that were independent of different dosing regimens. The data were described as the geometric means with 95% confidence interval using Stata version 16.1 (StataCorp, Texas, USA), unless otherwise specified. Non-overlapping 95% CI were used to indicate statistically significant differences between groups. This was an exploratory study, with post hoc analysis of samples from three separate trials, with different participants in each. Figures were produced using Prism 8 (GraphPad Software, California, USA).

## Results

**Fig. 1:**
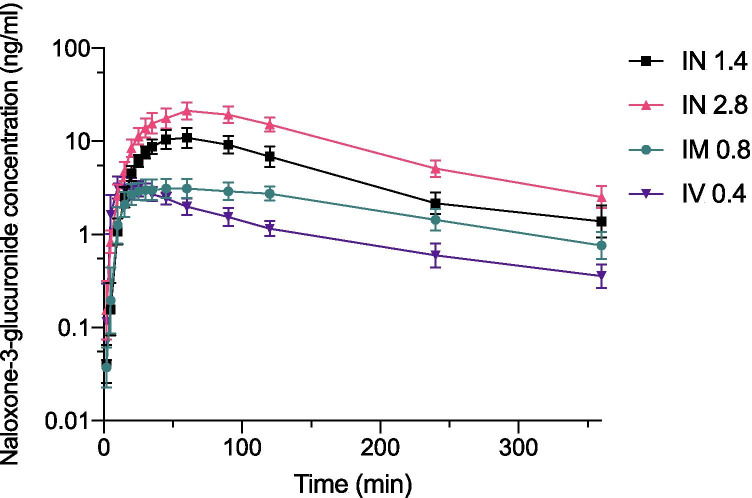
Change in serum concentrations of naloxone-3-glucuronide over time after administration of intranasal (1.4 mg and 2.8 mg), intramuscular (0.8 mg), and intravenous (0.4 mg) naloxone in healthy volunteers (n = 12) who were not exposed to remifentanil Samples were analysed in study III. Data are presented as the geometric means with 95% confidence intervals. Abbreviations: IN, intranasal; IM, intramuscular; IV, intravenous

Figure 1 presents the time course of N3G concentrations after administration of IV (0.4 mg), IM (0.8 mg), and IN (1.4 mg and 2 × 1.4 mg) naloxone hydrochloride (Naloxone B Braun, Melsungen, Germany). The most prominent observation (Table 1) was that the Tmax for the metabolite by parenteral naloxone administration was 9–17 for IV and 36–40 min for IM, compared to 59–71 min for IN naloxone. The dose-corrected Cmax of N3G (Table 1) after intranasal administration of naloxone under remifentanil exposure was significantly lower (4.5 ng/mL) than in subjects not exposed to remifentanil (7.8–8.4 ng/mL). This difference was not observed in IM and IV administration.

**Table 1:** Pharmacokinetic variables of naloxone-3-glucuronide after intranasal, intramuscular and intravenous administration of naloxone in healthy volunteers with and without remifentanil coadministration

Dose	Route	Remi	AUC_0-20_ (min⋅ng/ml)	Dose corrected AUC_0-20_ (min⋅ng/ml)	AUC_0-120_ (min⋅ng/ml)	Dose corrected AUC_0-120_ (min⋅ng/ml)	AUC_0-360_ (min⋅ng/ml)	Dose corrected AUC_0-360_ (min⋅ng/ml)	C_max_ (ng/ml)	Dose corrected C_max_ (ng/ml)	T_max_ (min)
0.8 mg [2]	IN	Yes	12 (8–17)	14 (10–21)	304 (238–390)	380 (297–487)	647 (472–885)	808 (590–1107)	3.6 (2.9–4.5)	4.5 (3.6–5.7)	71 (55–92)
1.4 mg [16]	IN	No	31 (25–38)	22 (18–27)	946 (793–1129)	676 (567–806)	1731 (1437–2086)	1237 (1027–1490)	12 (9.7–14)	8.4 (6.9–10)	59 (47–74)
2.8 mg [16]	IN	No	62 (50–77)	22 (18–28)	1846 (1520–2242)	659 (543–801)	3552 (2976–4241)	1269 (1063–1515)	22 (18–27)	7.8 (6.3–9.6)	66 (55–78)
0.8 mg [2]	IM	Yes	20 (13–29)	25 (16–37)	243 (179–330)	304 (224–412)	550 (426–711)	688 (532–889)	2.5 (1.9–3.4)	3.2 (2.3–4.2)	40 (33–49)
0.8 mg [16]	IM	No	25 (18–35)	32 (23–44)	325 (262–403)	407 (328–504)	720 (591–877)	899 (738–1096)	3.4 (2.7–4.4)	4.3 (3.4–5.5)	36 (24–55)
1.0 mg [15]	IV	Yes	121 (98–150)	121 (98–150)	493 (390–622)	493 (390–622)	-	-	7.7 (6.2–9.6)	7.7 (6.2–9.6)	9.4 (7.5–11.8)
0.4 mg [16]	IV	No	47 (35–63)	118 (88–158)	250 (203–307)	624 (508–768)	419 (351–500)	1047 (878–1249)	3.6 (2.9–4.4)	8.9 (7.2–11)	17 (13–22)

Data are presented as geometric mean (95% confidence intervals). Abbreviations: Remi: remifentanil coadministration, AUC_0-360_: area under the curve until 360 min, AUC_0-120_: area under the curve until 120 min, AUC_0-20_: area under the curve until 20 min, Cmax: maximum concentration, Tmax: time to maximum concentration. N = 12 in all groups, except 1.0 mg IV where n = 11

**Fig. 2:**
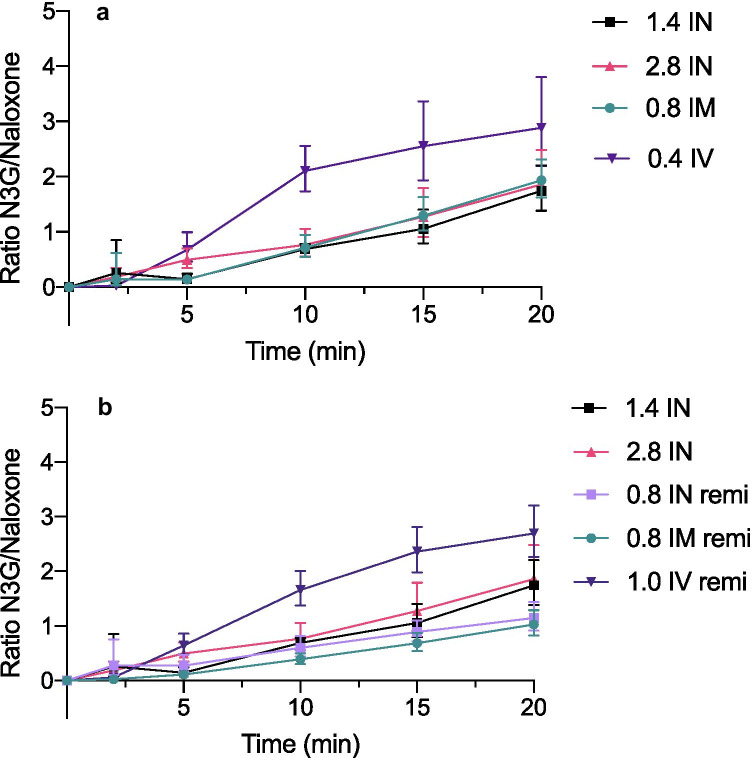
Change in the metabolite/naloxone ratio over 20 min in healthy volunteers, for data combined from three different studies 2a) Metabolite/naloxone ratio over the first 20 min after administration of intranasal (1.4 mg and 2.8 mg), intramuscular (0.8 mg), and intravenous (0.4 mg) naloxone in healthy volunteers (n = 12) who were not exposed to an opioid (study III). 2b) Metabolite/naloxone ratio over the first 20 min after administration of intranasal naloxone (1.4 mg and 2.8 mg) to healthy volunteers (n = 12) who were not exposed to an opioid (study III), combined with metabolite/naloxone ratio after intranasal naloxone (0.8 mg), intramuscular (0.8 mg), and intravenous naloxone (1.0 mg) in healthy volunteers who were exposed to the opioid remifentanil (study I and II). Data are presented as the geometric means with 95% confidence intervals. Abbreviations: IN, intranasal; IM, intramuscular; IV, intravenous

The dose-corrected N3G-AUC_0–20_ was higher after intravenous administration of naloxone than after intramuscular and intranasal administration (Table 1). Figure 2 displays the change in the ratio for N3G/naloxone over the first 20 min. As presented in Fig. 2a, the ratio increased more rapidly and reached higher levels after IV administration than after IM and IN administration, for which the curves were identical. As presented in Fig. 2b, N3G formation after IV naloxone under the influence of remifentanil followed the same pattern as in non-remifentanil exposure. The same pattern was observed for IM and IN; however, there may be a tendency toward lower ratios under remifentanil exposure after 15 min. The dose-corrected N3G-AUC_0–20_ for IN tended to be lower in the remifentanil-exposed group than in the non-exposed group (Table 1). The dose-corrected N3G-AUC_0–20_ after IV administration was considerably greater than that for IN administration, regardless of remifentanil exposure.

**Fig. 3:**
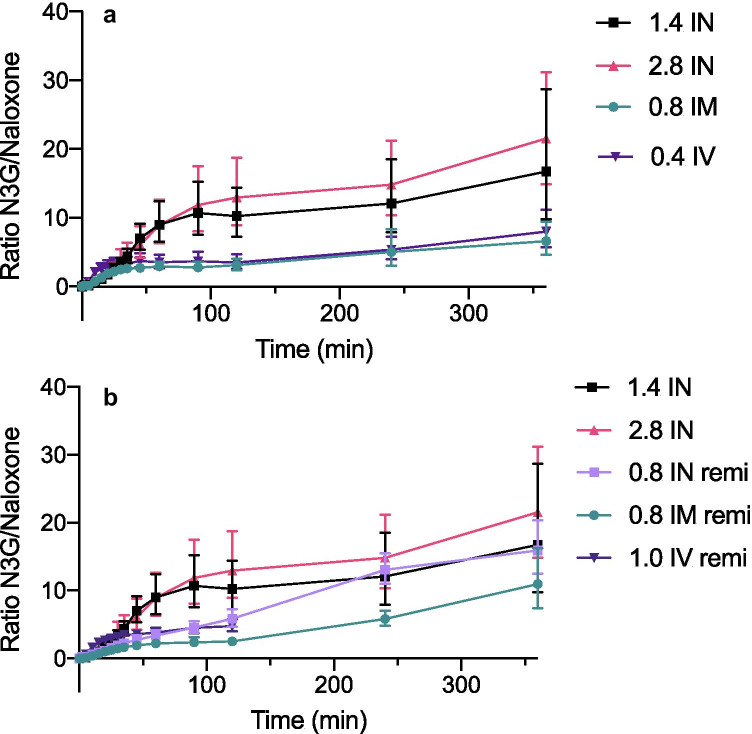
Change in the metabolite/naloxone ratio over 360 min in healthy volunteers, for data combined from three different studies 3a) Metabolite/naloxone ratio over the first 360 min after administration of intranasal (1.4 mg and 2.8 mg), intramuscular (0.8 mg), and intravenous (0.4 mg) naloxone in healthy volunteers (n = 12) who were not exposed to an opioid (study III). 3b) Metabolite/naloxone ratio over the first 360 min after administration of intranasal naloxone (1.4 mg and 2.8 mg) to healthy volunteers (n = 12) who were not exposed to an opioid (study III), combined with metabolite/naloxone ratio after intranasal naloxone (0.8 mg), intramuscular (0.8 mg), and intravenous naloxone (1.0 mg) in healthy volunteers who were exposed to the opioid remifentanil (study I and II). Data were only available for 120 min in the intravenous arm. Data are presented as the geometric means with 95% confidence intervals. Abbreviations: IN, intranasal; IM, intramuscular; IV, intravenous

Figure 3 displays the change in the metabolic ratio of N3G/naloxone up to 360 min. As presented in panel 3a, intranasal administration without remifentanil administration resulted in a clear change in the metabolic ratio compared to intramuscular and intravenous administration. The ratios after IN administration rose quickly after 30–90 min, with 2–3 times higher ratios after 360 min than after IV and IM administration.

The lower panel (Fig. 3b) indicates that remifentanil exposure along with intranasal administration results in a significant change with a considerably slower increase in the N3G/naloxone ratio. After the remifentanil infusion was discontinued at 90 min, this effect diminished gradually, and from 240 min onwards, there was no significant difference in the ratio following intranasal naloxone with or without co-administration of remifentanil. For IV (0–120 min) and IM (0–360 min) naloxone with remifentanil exposure, the levels were stable for up to 120 min; however, the ratio increased somewhat at 360 min after IM naloxone. This trend corresponds (Table 1) to that of the dose-corrected AUC_0–120_ with IN administration under remifentanil exposure, which was significantly lower than the dose-corrected value with IN administration without the opioid.

## Discussion

The major finding from this study was that there were no signs of nasal metabolism of naloxone. However, there was unequivocal evidence of the significantly increased pre-systemic formation of the metabolite N3G following nasal compared to intramuscular administration. Remifentanil appeared to reduce the formation of N3G after nasal administration of naloxone.

The major reason for rejecting the hypothesis of a substantial pre-systemic nasal metabolism was that there was no difference in the metabolic ratios within the first 20 min after nasal administration compared to IM naloxone administration. If nasal metabolism had been important, a substantial contribution of metabolite production had been expected during this time window, as it is generally agreed that the residence time of xenobiotics in the nasal cavity is limited to 15–30 min due to the continuous mucociliary transport towards the pharynx [20]. Secondly, the Tmax of the mother substance naloxone after nasal administration was approximately 20 min [12, 13, 16], which should secure satisfactory amounts of the substrate to allow for a significant local metabolism in that time period.

The metabolic ratios (N3G/naloxone) after intranasal administration started to differ from the corresponding values with parenteral administration after approximately 30–45 min, being higher for the rest of the 360 min period. This pattern, along with delayed formation of N3G, may indicate that the formation of N3G was due to the uptake of naloxone through the oral route after initial nasal administration due drugs being transported from the nasal cavity to the pharynx, oesophagus and stomach. The involvement of such an oral component from swallowed drug in metabolism has recently been shown for nasally administered esketamine [21]. The nasal bioavailability of naloxone is approximately 50%, and the rest of the nasal naloxone is not accounted for. The suggestion of an oral component from swallowed naloxone is supported by our data on the time to the maximum concentration of N3G. After IM administration, we found that the Tmax of N3G was 36 min, close to the Tmax of naloxone of 30 min after IM administration in human volunteers [22]. While after intranasal administration of naloxone, there was a significant delay in the Tmax of N3G to about 60 min, compared to a Tmax of IN naloxone that is 15–30 min [1]. This conforms with the delay that could be expected from a swallowed component responsible for the increased N3G formation, resulting in higher metabolic ratios.

Remifentanil reduced both the dose-corrected N3G-AUC_0–120_ and N3G-Cmax of the metabolite after the administration of nasal naloxone. This was not the case after IM and IV administration. Nevertheless, the N-AUC and N-Cmax of naloxone increased under remifentanil exposure, resulting in increased bioavailability after nasal administration from 50 to 75% [2]. The absolute oral bioavailability of naloxone is low, approximately 2% [5], and is sensitive to the inhibition of naloxone metabolism in the gastrointestinal tract or the liver. Thus, the increased bioavailability of naloxone after nasal administration during remifentanil infusion may be explained by a higher oral bioavailability of swallowed naloxone due to reduction of the pre-systemic metabolism of naloxone by remifentanil. For nasal esketamine it was shown that a decrease in hepatic blood flow gave an increase in AUC and Cmax of esketamine [21]. Reduced portal blood flow is a common effect of many sedative drugs [23], and could be the explanation of a potential interaction between remifentanil and nasal naloxone. Our observations were from studies employing the opioid remifentanil. However, as the effect on portal flow is general for many sedatives, that also could include other opioids. If the same effect exists for other opioids such as heroin and fentanyl, which are the major culprits of opioid overdoses in the community, this could increase the exposure to the opioid antagonist after nasal naloxone in overdose patients compared to the use of IM or IV routes. A possible interaction between remifentanil or other opioid agonists with naloxone must also be accounted for when interpreting results obtained from previous pharmacokinetic studies in healthy volunteers, and in the planning of future trials.

Future opioid antagonist products such as nalmefene nasal spray are in the pipeline [24]. These products should be studied in volunteers or patients with co- administration of opioids, preferably those drugs causing overdoses in the community. Interactions that increase the potency of antagonism may also increase the propensity for opioid withdrawal. This is not a trivial matter, but an avoidable iatrogenic harm.

This study has several limitations. We used data from several different studies in which different naloxone doses were used. Due to resource constraints, we could only analyse 12 of the 22 participants in one of the studies [16]. To render data comparable across different studies, two strategies were used. First, the metabolic ratio of metabolite to mother substance, N3G/naloxone provided figures that were independent of the dose. Second, dose-corrected AUC and Cmax values for N3G were used to circumvent the problem with different doses. Similar studies establishing any interaction between nasal naloxone or other antagonists and opioids common in overdose is needed. Third, the nature of the study material did not allow for formal statistical testing.

## Conclusion

The pre-systemic metabolism of naloxone after nasal administration does not occur in the nose; it is mediated by an oral component of swallowed medication present in the gut. Remifentanil increases the bioavailability of naloxone after nasal administration by reducing the pre-systemic metabolism of this oral component of the nasally administered drug. If the same effect exists for other opioids more common in overdoses in the community this could increase the exposure to naloxone in patients and prolong the effect of nasally administered naloxone compared to the expectations from results obtained from pharmacokinetic studies in healthy volunteers.

## Supplementary Information

Below is the link to the electronic supplementary material.

Supplementary file1 (PDF 233 KB)

## Data Availability

Supporting data is not available as participants of these studies did not agree for their data to be shared publicly.
