# Supplementary material for: The pharmacokinetic interaction between nasally administered naloxone and the opioid remifentanil in human volunteers
Source: Eur J Clin Pharmacol. 2021 Jul 29;77(12):1901–8. doi: 10.1007/s00228-021-03190-1 (PMC8585821; doi:10.1007/s00228-021-03190-1)
Supplement: Supplementary file 1 — Supplementary file1 (PDF 233 KB) [file 228_2021_3190_MOESM1_ESM.pdf]

NTNU
Norwegian University of Science and Technology

Supplementary material to:

The mechanism of the pharmacokinetic interaction between nasally administered naloxone and the opioid remifentanil in human volunteers

Authors

Ida Tylleskar MD, PhD, Sissel Skarra BSc, Arne Kristian Skulberg MD, PhD, Ola Dale MD, PhD

Correspondance:

Dr. Ida Tylleskar
Department of Circulation and Medical Imaging, NTNU
Post-box 8905, 7491 Trondheim, Norway
Email: ida.tylleskar@ntnu.no

Telephone: +47 45449894

Table of contents

1 DESCRIPTION OF THE ORIGINAL DRUG TRIALS AND DRUG ANALYSIS 2
1.1 SUBJECTS 2
1.2 STUDY DESIGNS 3
1.3 TREATMENTS 4
TABLE S1. DESIGN OF STUDY I-III 4
1.4 PHARMACODYNAMIC STUDY DESIGN 5
TABLE S2. SUMMARY OF THE CLINICAL TRIALS USED TO GATHER DATA ON THE METABOLITE NALOXONE-3-GLUCURONIDE 5
1.5 BLOOD SAMPLING 6
2 DETERMINATION OF DRUG CONCENTRATIONS OF NALOXONE AND NALOXONE-3-GLUCURONIDE 7
3 ADDITIONAL AUC INFORMATION 8
TABLE S3. AREAS UNDER THE CURVE FOR NALOXONE WITH AND WITHOUT REMIFENTANIL INFUSION FROM FOUR DIFFERENT STUDIES IN HEALTHY VOLUNTEERS 8
4 REFERENCES 8

1 Description of the original drug trials and drug analysis

Samples from three studies of naloxone in human healthy volunteers have been included in secondary analyses of the metabolite naloxone-3-glucuronide (naloxone-3-β-D-glucuronide). All studies were conducted according to the guidelines for Good Clinical Practice and Ethical Principles for Medical Research Involving Human Subjects outlined in the Declaration of Helsinki. The original studies (study I: Skulberg et al., 2018 [1], study II: Tylleskar et al., 2018 [2], study III: Skulberg et al. 2019 [3]) were all approved by The Regional Committees of Medical and Health Research Ethics (2014/740, 2014/2194, 2015/1285), the Norwegian Medicines Agency (EudraCT 2014-001465-27, 2014–005348-16, 2015–002355-10), and were also in clinicaltrials.gov (NCT02307721, NCT02405988, NCT02598856). The clinical trials were conducted at Clinical Trials Units at St. Olavs Hospital, Trondheim between February 2014 and September 2016, and for study III there were also a study site at Oslo University Hospital (Rikshospitalet), Oslo, Norway between October 2015 and September 2016 . Participants were insured trough the Drug Liability Association, Norway.

1.1 Subjects

Twelve healthy volunteers participated in each of study I and II. In study III, 22 participants participated in the study and 12 of these were randomly selected for analysis of naloxone-3-glucuronide after the study had been completed. In all, 36 healthy volunteers (18 men, 18 women) contributed with samples from three separate studies. The study designs and the summary of the studies are shown in supplementary tables S1 and S2. Healthy men and women aged 18–40 were eligible for inclusion. Before screening subjects were given information about the study and signed a written informed consent.

Inclusion criteria were ASA class I (medical history, clinical examination, ECG without pathological findings and with laboratory tests including haemoglobin, creatinine, alanine aminotransferase, aspartate aminotransferase and gamma-glutamyltransferase within reference values). A body mass index within 18.5- 24.9 (study I) and 18.5- 26.0 kg/m2 (study II and III) was requiered. Women with childbearing potential had to use high efficacy contraception throughout the study until the last visit.

Exclusion criteria in all the studies were:

- Subjects using medication on a regular basis, including regular use of nasal spray of any form
- History of prior drug allergy
- Hypersensitivity to naloxone or any of its excipients

- Subjects having local nasal disease or nasal surgery for the last 2 months
- Pregnant or breastfeeding women. A serum HCG below 3 U/L must be demonstrated in women of childbearing potential at screening visit
- Current or history of drug or alcohol abuse
- Have received another new medical chemical entity (defined as a compound which has not been approved for marketing) or have participated in any other clinical study that included drug treatment within 3 months of the administration of investigational product in this study (only for study III).
- Investigator considers subject unlikely to comply with study procedures, restrictions and/ or other requirements.

For study I and II the following exclusion criteria were also applied as the subjects were given remifentanil:

- Participants with a history of contact with police or authorities in relation to alcohol or drug offences
- A history of prolonged use of opioid analgesics
- Participants who had access to remifentanil or other potent opioids in their workplace
- Previous participation in trials where they received opioids
- Subjects who had a history of drug and/or alcohol abuse were excluded.

Potential participants had to answer the CAGE-AID questionnaire, and subjects answering yes to two or more questions were excluded. In study II they also had to pass the Allen's test of collateral circulation of the hand, and blood donation 6 weeks before and after study participation was not allowed.

1.2 Study designs

All studies were open, randomised, controlled cross-over trials, except study II that was a study with only one treatment arm. The studies were conducted during 2014 – 2016 at the Clinical Research Facility, St. Olavs hospital, Trondheim University Hospital, Norway. Study III also had a study centre at Rikshospitalet, Oslo, Norway.

Each study session lasted for 6–7 hours and the sessions were separated by at least 72-hours wash-out periods, in all studies except study II having a three-hours study session only. Subjects had to abstain from all medications for 7 days before treatment. No fasting or other meal restrictions were required in study III. In study I and II the participants were exposed to a remifentanil infusion, and a pre-intervention 6-hours fasting regime was required.

Naloxone was given intranasally (IN), intravenously (IV) and intramuscularly (IM) in various doses. The doses and administration routes of naloxone for the different studies are described in table S1. The order of treatment allocation was decided by randomisation. This was performed in a concealed fashion by an internet-based service.

1.3 Treatments

Naloxone was given as naloxone hydrochloride. Dose and route of naloxone administration for the different studies are shown in table S1. All medications were weighed before and after delivery for determination of the exact doses.

Nasal naloxone for study I were delivered by Department of Biopharmaceutical Production, Norwegian Institute of Public Health (FHI), Oslo, Norway. Nasal sprays for study III were manufactured by AS Den norske Eterfabrikk, Oslo, Norway. A disposable nasal spray device from Aptar Pharma (Louveciennes, France), the "Aptar Unitdose" device was used for delivering the nasal spray. The device delivered 0.1 ml per actuation. The formulation was tested with different concentrations of naloxone hydrochloride: 8 mg/ml and 14 mg/ml. The nasal formulation is previously published [4].

The Hospital Pharmacy at St Olavs hospital, Trondheim University Hospital delivered Naloxon B. Braun 0.4 mg/ml (Melsungen, Germany) for intravenous and intramuscular administration. The intravenous injections were administrated as a 1.0 – 2.5 ml rapid bolus. Intramuscular injections were administered as 2.0 ml bolus in the deltoid muscle.

Table S1. Design of study I-III

Study	Naloxone		Remifentanil	Sampling time	Reference
	Route	Dose (mg)	TCI plasma target (ng/ml)	(hours)	
I	IN<br>IM	0.8<br>0.8	1.0<br>1.3<br>2.5	6 h	[1]
II	IV	1.0	1.3	2 h	[2]
III	IN<br>IN<br>IM<br>IV	1.4<br>2×1.4*<br>0.8<br>0.4	NA	6 h	[3]

IN = intranasal, IV = intravenous, IM = intramuscular, TCI = target controlled infusion, NA *= not applicable*
** Given as 2 doses of 1.4 mg in the same nostril. The doses were separated by 3 minutes.*

1.4 Pharmacodynamic study design

Study I and II were pharmacokinetic-pharmacodynamic studies. To assess the effects of naloxone it must be administered to subjects under influence of an opioid. The subjects were given an intravenous infusion of remifentanil. It was given by plasma target controlled infusion (TCI), Minto model using Alaris PK Guardrail pumps (CareFusion Cooperation, UK). Three different targets were used in study I: 1.0 (*n*=3), 1.3 (*n*=5) and 2.5 ng/ml (*n*=4). The same participant had the same target on both study days. In study II the plasma target was 1.3 ng/ml for all the participants. The infusion was given for 12 minutes before naloxone was administrated. The infusion was discontinued 90 minutes after naloxone administration. The total infusion time was therefore 102 minutes.

The effect of the opioid and the antagonist were measured with pupillometry, a well-recognised measure of opioid effect in experimental studies in man. The measurements were conducted using a Neuroptics VIP 200 Pupillometer (Neuroptics, Irvine, CA, USA), and the participants were asked to focus on a distant point in the room during the measurements. The participants were kept in a room with stable ambient lighting, and a luxometer was used to ensure similar light conditions in each visit of each participant.

Table S2. Summary of the clinical trials used to gather data on the metabolite naloxone-3-glucuronide

Study	n	n samples/ session	Sex (f/m)	Age (yrs*)	Weight (kgs*)	Naloxone dose (mg)	Route	Remifentanil plasma target TCI	Sampling period
I [1]	12	15	6/6	24<br>(21–27)	66.3<br>(51.9– 95.0)	0.8<br>0.8	IN<br>IM	1.0<br>1.3<br>2.5	6 h
II [2]	12	13	6/6	23<br>(19–26)	67.1<br>(55.4–88.9)	1.0	IV	1.3	2 h
III [3]	12	15	6/6	25.0<br>(21.4-27.3)	73.1<br>(61.1-90.3)	1.4<br>2 × 1.4<br>0.8<br>0.4	IN<br>IN<br>IM<br>IV	NA	6 h

f = *female*, m = *male*, IN = *intranasal*, IV = *intravenous*, IM = *intramuscular*, TCI = *target controlled infusion*, NA = *not applicable*

* *Given as median and (min-max)*

1.5 Blood sampling

Every study day a venous cannula for blood sampling was placed in the antecubital fossa, and participants were monitored for oxygen saturation and non-invasive blood pressure for safety. For the studies with intravenous administration of medications, a separate venous cannula was placed for this purpose. Venous blood samples were taken prior to naloxone administration and at 2, 5, 10, 15, 20, 25, 30, 35, 45, 60, 90, 120, 240, and 360 min after naloxone administration. For study II, the last sample was taken at 120 minutes.

In studies I-II serum was used to analyze naloxone and the main metabolite naloxone-3-glucuronide. Plasma (K_2EDTA) was used for these analyses in study III.

2 Determination of drug concentrations of naloxone and naloxone-3-glucuronide

Naloxone was analysed by a validated high performance liquid chromatography tandem mass spectrometry method at the Proteomics and Modomics Experimental Core Facility (PROMEC), Norwegian University of Science and Technology (NTNU), Norway. The main naloxone metabolite, naloxone-3-glucuronide, was analysed alongside naloxone. The analysis were performed on a LCMSMS system, AB Sciex Triple Quad 5500, operating in positive mode. The method is published in full [4].

The reference materials for naloxone, the metabolite naloxone-3-β-D-glucuronide and the internal standard naloxone-d5 were purchased from Sigma-Aldrich (St. Louis, MO, USA). The internal standard naloxone-d5 3-β-D-glucuronide was from Toronto Research Chemicals Inc.. Sample analysis were performed by multiple reaction mode. Ion pairs were 328.2/268.2 and 333.2/273.2 for naloxone and the internal standard. For naloxone-3-glucuronid and its internal standard, the ion pairs were 504/310.1 and 509/315.2.

Calibration range for naloxone was 0.02 – 10 ng/ml (study I), 0.02 – 45 ng/ml (study II) and 0.02 – 50 ng/ml (study III). The limit of quantitation (LOQ) was 0.02 ng/ml for all three studies, with a mean coefficient of variation (CV) < 5.1 % and inaccuracy < 3.6 %, n = 31.
Quality controls for naloxone in the three studies were QC1 (0.05 ng/ml), QC2 (3.0 ng/ml), QC3 (15 ng/ml) and QC4 (30 ng/ml). In total the in-run CV and inaccuracy were < 5.5 %, < 4.8 % (QC1, n = 30), < 4.4 %, < 8.5 % (QC2, n = 24), < 7.4 %, < 5.6 % (QC3, n = 33) and < 7.0 %, < 6.6 % (QC4, n = 32), respectively.

Calibration range for the metabolite, naloxone-3-glucuronide, was 0.02 – 45 ng/ml with 9 calibration standards for the studies I, II and III. LOQ was 0.05 ng/ml with CV < 5.9 % and inaccuracy < 1.7 %, n = 24. Quality controls for the metabolite were QC1 (0.1 ng/ml), QC2 (3.0 ng/ml), QC3 (14 ng/ml) and QC4 (30 ng/ml). In the pre-run validation CV and inaccuracy were < 7.1 %, < 0.7 % (QC1, n = 24), < 7.5 %, < 5.6 % (QC2, n = 6), < 4.1 %, < 1.8 % (QC3, n = 18), < 3.6 %, < 0.4 % (QC4, n = 24). During in-run validation CV and inaccuracy were < 6.4 %, 0.4 % (QC1, n = 54), < 4.8 %, < 6.4 % (QC2, n = 26), < 4.4 %, < 0.2 % (QC3, n = 28), < 4.6 %, < 0.0 % (QC4, n = 51), respectively.

3 Additional AUC information

Table S3. Areas under the curve for naloxone with and without remifentanil infusion from four different studies in healthy volunteers

	Intranasal naloxone 0.8 mg		Intramuscular naloxone 0.8 mg		Intravenous naloxone 1.0 mg	
	Without opioid [4]	With Remifentanil [1]	Without opioid [3]	With Remifentanil [1]	Without opioid [4]	With Remifentanil [2]
**AUC_{0-120}**	65 (50-80)	114 (92-136)	118 (106 -130)	167 (132-202)	194 (163-223	219 (183-256)
**AUC_{0-360}**	99 (77-121)	160 (125-195)	185 (170-201)	167 (132-202)	NA	NA

Area under the curve for 0-120 minutes (AUC_{0-120}) and for 0-360 minutes (AUC_{0-360}) for naloxone with and without remifentanil infusion. Data are given as mean (95% confidence interval). The data are compiled from four different studies in healthy volunteers (see reference). NA = *not applicable*

4 References

1. Skulberg AK, Tylleskar I, Nilsen T, Skarra S, Salvesen Ø, Sand T, Loftsson T, Dale O (2018) Pharmacokinetics and -dynamics of intramuscular and intranasal naloxone in healthy volunteers. Eur J Clin Pharmacol 74 (7):873-883. doi:10.1007/s00228-018-2443-3
2. Tylleskar I, Skulberg AK, Skarra S, Nilsen T, Dale O (2018) Pharmacodynamics and arteriovenous difference of intravenous naloxone in healthy volunteers exposed to remifentanil. Eur J Clin Pharmacol 74 (12):1547-1553. doi:10.1007/s00228-018-2545-y
3. Skulberg AK, Asberg A, Khiabani HZ, Rostad H, Tylleskar I, Dale O (2019) Pharmacokinetics of a novel, approved, 1.4-mg intranasal naloxone formulation for reversal of opioid overdose-a randomized controlled trial. Addiction 114 (5):859-867. doi:10.1111/add.14552
4. Tylleskar I, Skulberg AK, Nilsen T, Skarra S, Jansook P, Dale O (2017) Pharmacokinetics of a new, nasal formulation of naloxone. Eur J Clin Pharmacol 73 (5):555-562. doi:10.1007/s00228-016-2191-1
